# Secondary syphilis presenting as fever of unknown origin

**DOI:** 10.1002/ccr3.8583

**Published:** 2024-03-08

**Authors:** Hannah Eloise Wilding, Amy Hays

**Affiliations:** ^1^ Penn State College of Medicine Hershey Pennsylvania USA; ^2^ Department of Family and Community Medicine Penn State College of Medicine Hershey Pennsylvania USA

**Keywords:** communicable diseases, fever of unknown origin, syphilis, *Treponema pallidum*

## Abstract

A thirty‐eight year‐old male presented with a seven‐week history of persistent fever accompanied by recurrent night sweats, chills, arthralgias, headache, and chest tightness.Initial laboratory testing showed non‐specific elevation of inflammatory markers, but was otherwise unremarkable.A palmar rash developed one week later, prompting testing for syphilis. Fluorescent treponemal antibody absorption (FTA‐ABS) and rapid plasma reagin (RPR) tests were both positive.Penicillin G was administered and the patient recovered uneventfully.Our case emphasizes the need for increased syphilis screening to ensure proper diagnosis and prompt treatment.

## Graphical abstract

Image of patient's maculopapular syphilitic rash on palmar surface of his hand, which developed about 8 weeks into his fever.

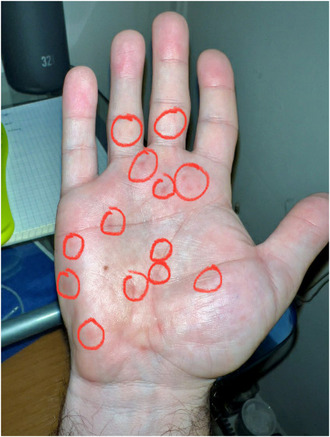

## 1 INTRODUCTION

Fever of unknown origin (FUO) is a challenging diagnostic dilemma. Defined as a fever of 100.9°F (38.3°C) or higher lasting at least 3 weeks with an unrevealing diagnostic work up,^1^ FUO's in adults typically represent unusual presentations of common illnesses. Most often, the condition resolves without a specific diagnosis being made.^2^

The differential diagnosis for FUO is extensive. A helpful approach is to consider infectious, inflammatory, and malignant diseases, as well as miscellaneous conditions such as thyroiditis, thromboembolic disease, and factitious fever. A comprehensive history emphasizing social and travel components should precede a complete physical examination, with laboratory tests and imaging guided by pertinent findings on history and examination.

## 2 CASE HISTORY/EXAMINATION

**FIGURE 1:**
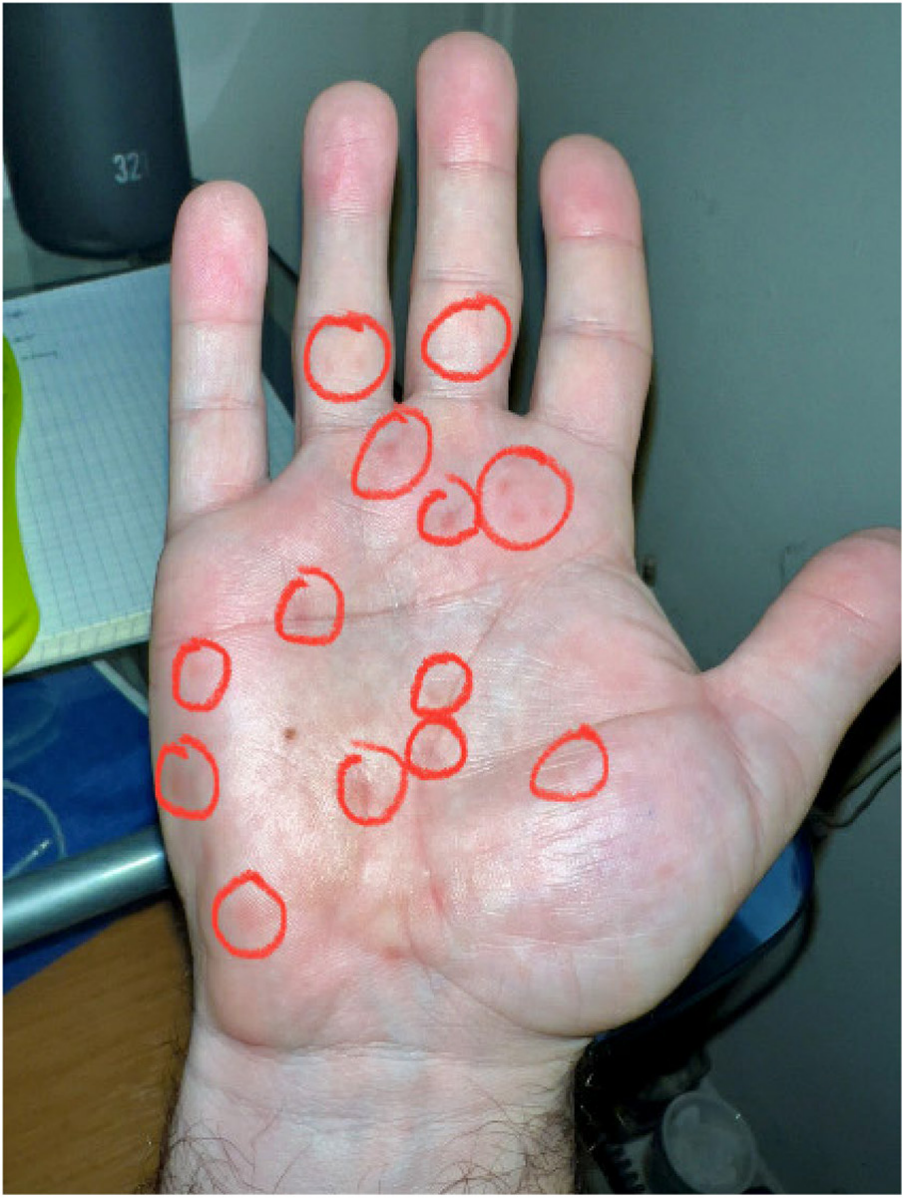
Image of patient's maculopapular syphilitic rash on palmar surface of his hand.

A 38 year‐old male with a past medical history (PMH) of anxiety, depression, chronic back pain, obesity, irritable bowel syndrome, and dyshidrotic eczema, presented to the outpatient primary care clinic with a seven‐week history of fever unresponsive to oral antipyretics. The patient also noted recurrent chills, night sweats, myalgias, headaches, and chest tightness. Three days before presentation, he developed a cough with clear sputum. A complete review of systems was otherwise negative. A careful history emphasizing surgical, social, and travel components revealed that the patient had undergone arthroscopic ACL reconstruction 7 weeks prior to presentation, after which his fever began. He tested negative for COVID‐19 preoperatively. On surgical follow‐up, his orthopedist found no surgical or thromboembolic complications or other iatrogenic cause for fever. Approximately 1 week after initial presentation, the patient developed an erythematous maculopapular rash on the palmar surfaces of both hands (Figure 1). Arthralgias, myalgias, and morning stiffness worsened, and the rash spread over his torso.

Regarding other medical history, his substance use history included one cigarette per day for the prior 12 years. His exercise regimen was walking about four to five times per week. Medications included acetaminophen‐oxycodone, lorazepam as needed, and bupropion. His family history included Alzheimer disease in his paternal uncle and paternal grandmother, breast cancer and hypertension in his mother, ankylosing spondylitis in his sister, and prostate cancer in his paternal grandfather. He was sexually active with one consistent male partner.

### 2.1 Differential diagnosis, investigations, and treatment

Differential diagnosis included COVID‐19, influenza A and B, Hepatitis C, HIV, tickborne illnesses, postoperative wound infection, thromboembolic disease, and infectious endocarditis. Tickborne illnesses, particularly Lyme disease and Anaplasmosis common in the geographical area where the patient lives, were excluded by serology. Postoperative wound infection and thromboembolic disease were not evident clinically. The lack of objective synovitis on examination made rheumatological disease less likely. Malignancy was considered in light of the complaint of night sweats, but there was no lymphadenopathy or weight loss.

**FIGURE 2:**
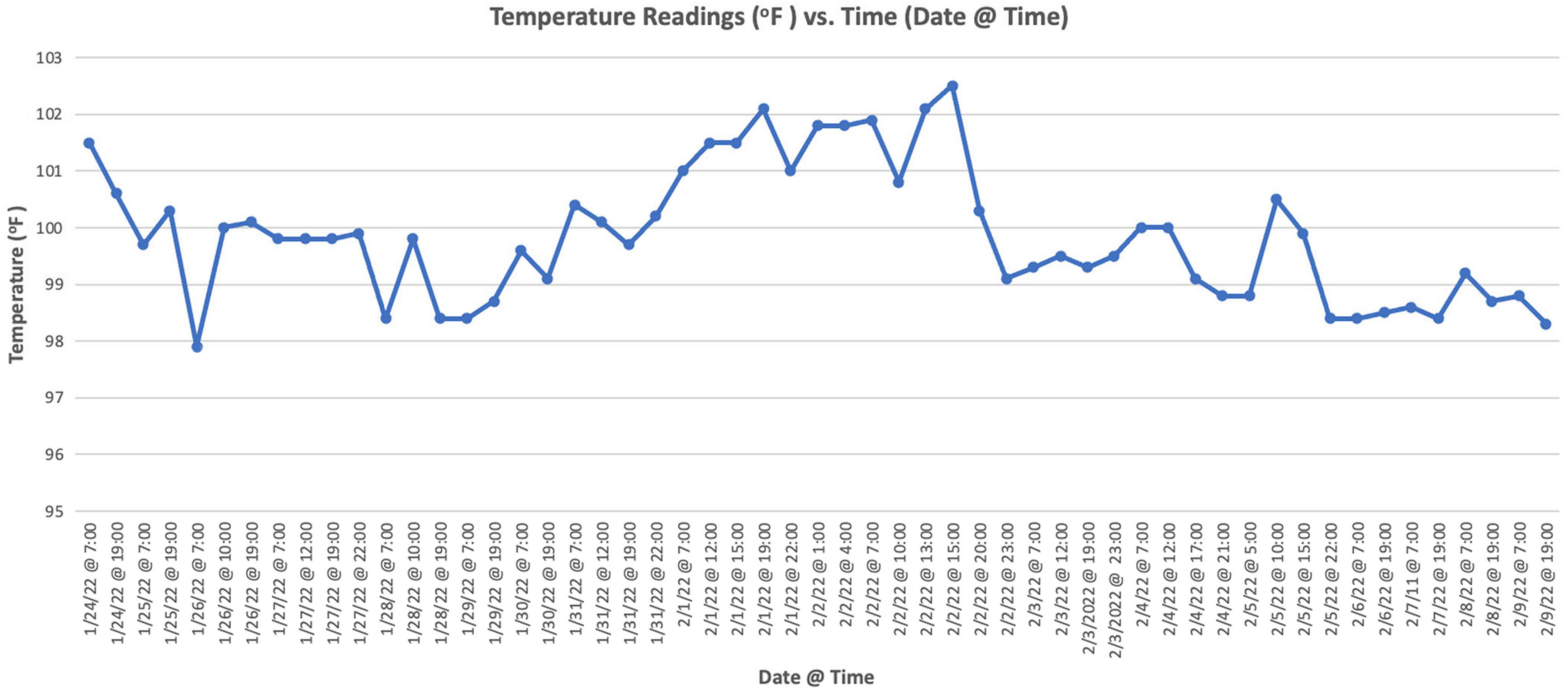
Graph of patient's temperature readings (°F) and corresponding dates and times of measurement from January 24, 2022 to February 09, 2022.

The patient was asked to record daily body temperatures for 3 weeks (Figure 2). Complete blood count, comprehensive metabolic panel, liver function tests, thyroid stimulating hormone, erythrocyte sedimentation rate, C‐reactive protein, chest x‐ray, urinalysis, COVID‐19 test, and influenza A and B tests were performed. Additionally, titers for Lyme disease, Anaplasmosis, Hepatitis C, and HIV were obtained. All were normal or negative, with the following exceptions: lymphocyte count was low at 780/mL, monocyte count was elevated at 1302/mL, inflammatory markers were increased, and 2+ ketones and trace protein were found on urinalysis. Elevation of inflammatory markers was perceived to be a nonspecific finding. Abnormal laboratory results are listed in Table 1, while normal laboratory results are listed in Table S1.

**TABLE 1:** Positive results of laboratory workup initiated for fever of unknown origin.

CRP High Sensitivity (mg/L)	>10.0 (High)
ANA Screen	Positive
ESR (mm/h)	92 (High)
Absolute lymphocytes (cells/μL)	780 (Low)
Absolute monocytes (cells/μL)	1302 (High)
Urine ketones	2+
Urine protein	Trace
RPR Screen	Reactive
RPR Titer	1:32 (High)
FTA‐ABS	Reactive

Abbreviations: ANA, antinuclear antibody; CRP, C reactive protein; FTA, fluorescent treponemal antibody absorption test; SR, erythrocyte sedimentation rate; RPR, rapid plasma reagin.

At the time of development of palmar rash, antinuclear antibody titer (ANA) was ordered. Three sets of blood cultures (obtained on different days) and transthoracic echocardiogram were also performed to exclude infectious endocarditis. The ANA titer was positive, but all other studies were normal. As the rash spread, fluorescent treponemal antibody absorption test (FTA‐ABS) and rapid plasma reagin (RPR) test were ordered, both returning positive. Accordingly, secondary syphilis was diagnosed. He had no PMH of non‐venereal treponemal disease. He also had no known history of similar symptoms or other sexually transmitted infections (STI). HIV antigen/antibody screening was negative, but other STI workup was not performed since patient was asymptomatic for such conditions, hence suspicion for his infection being sexually transmitted was low on initial presentation.

The patient was treated with Penicillin G benzathine 2.4 million units IM as an outpatient. Post‐injection, the patient experienced a transient flare of his rash, consistent with Jarisch–Herxheimer reaction. The rash was self‐limited and resolved after approximately 72 hours.

### 2.2 Outcome and follow‐up

Following treatment, the patient defervesced and all skin lesions resolved within about 1 week. Despite his clinical improvement, the patient noted depressive symptoms and attributed these to conflict with his partner that resulted from the diagnosis of syphilis. Ten months later, follow up RPR and HIV were negative, and the symptoms of depression had resolved.

## 3 DISCUSSION

Syphilis is caused by the motile gram‐negative spirochete *Treponema pallidum*. It has been described as the “great imitator,” due to its invasive and immunoevasive nature.^3^ It is transmitted sexually or vertically during pregnancy. In the 1990's, syphilis made a resurgence worldwide, most prominently in men who have sex with men (MSM). In 2015, the United States case rate for primary and secondary syphilis in MSM was 221 times the rate for women and 106 times the rate for heterosexual males.^3^ The World Health Organization (WHO) estimated that 17.7 million people between ages 15 and 49 had syphilis in 2012, with 5.6 million new cases yearly.^3^

*Treponema pallidum* is an obligate human pathogen, which enters the body through areas of microtrauma. Primary syphilis occurs approximately 2–3 weeks after initial infection, and is evidenced by the development of the chancre. This phase of illness may pass unnoticed, thereby enabling progression of infection.^4^ Approximately 4–8 weeks after the primary stage, secondary syphilis presents as bacteremia associated with a widespread maculopapular rash which may involve the scalp, palms, and soles of the feet. Condyloma lata and systemic symptoms may also be present. Late latent syphilis, or tertiary syphilis, occurs in approximately 35% of untreated patients, though this is seen most commonly in resource‐poor nations.^4^ Tertiary syphilis may manifest as cardiovascular syphilis, neurosyphilis, or gummatous syphilis.^4^

The incidence of syphilis in the United States has gradually increased over the last 20 years. The Centers for Disease Control and Prevention (CDC) reported an increase in primary and secondary cases, from 5973 in 2000 to 30,644 in 2017.^5^ This resurgence may result from unprotected sexual intercourse, multiple sexual partners, MSM, the popularity of online dating apps, intravenous drug use, and HIV co‐infection.^5^ Accordingly, the CDC recommends universal screening for syphilis in pregnancy. Testing protocols for high‐risk patients, including incarcerated populations, sex workers, MSM, patients with HIV, and transgender/gender‐diverse people have also been developed.^6^, ^7^

In summary, syphilis is an uncommon etiology for fever of unknown origin. Evidence‐based clinical resources, including UpToDate and American Family Physician, do not thoroughly discuss syphilis in the differential diagnosis of FUO.^1^, ^2^, ^8^ Extensive literature search reveals minimal and largely outdated case reports for this duo.^9^, ^10^ In such case reports, syphilis was similarly not discovered as the FUO etiology until multiple weeks of clinical workup.

Given this increasing incidence of syphilis, physicians need to remain vigilant. In addition, the stigma associated with this diagnosis may make patients reticent to provide relevant historical data. When considering the risk factors for syphilis, many patients belonging to these stigmatized populations are also medically underserved. Physicians bear the responsibility of investigating this diagnosis, while obtaining a history in a patient‐sensitive manner. Treatment for this condition is simple and effective, whereas the sequelae of untreated syphilis may be catastrophic.

## AUTHOR CONTRIBUTIONS

**Hannah Eloise Wilding:** Conceptualization; writing – original draft; writing – review and editing. **Amy Hays:** Conceptualization; supervision; writing – review and editing.

## FUNDING INFORMATION

The authors have nothing to disclose.

## CONFLICT OF INTEREST STATEMENT

The authors have no conflicts of interest to disclose.

## CONSENT

Written informed consent was obtained from the patient to publish this report in accordance with the journal's patient consent policy.

## Supporting information


Table S1.

## Data Availability

Data sharing not applicable to this article as no datasets were generated or analysed during the current study.
