# Supplementary material for: Secondary syphilis presenting as fever of unknown origin
Source: Clin Case Rep. 2024 Mar 8;12(3):e8583. doi: 10.1002/ccr3.8583 (PMC10923698; doi:10.1002/ccr3.8583)
Supplement: Supplementary file 1 — Table S1. [file CCR3-12-e8583-s001.docx]

**Supplemental Table 1:** Negative or Unremarkable Results of Laboratory Workup for Fever of Unknown Origin

| Na (mmol/L) | 138 |
| --- | --- |
| K (mmol/L) | 4.2 |
| Cl (mmol/L) | 100 |
| CO_2_ (mmol/L) | 24 |
| BUN (mg/dL) | 17 |
| Creatinine (mg/dL) | 0.88 |
| Estimated GFR (mL/min/1.73 m^2^) | 109 |
| Glucose (mg/dL) | 86 |
| Calcium (mg/dL) | 9.5 |
| ALT (units/L) | 35 |
| Total Bilirubin (mg/dL) | 0.6 |
| Alkaline Phosphatase (units/L) | 90 |
| AST (units/L) | 15 |
| Hepatitis C antibody | Non-reactive |
| Albumin (g/dL) | 4.4 |
| Protein (g/dL) | 7.7 |
| Globulin (g/dL) | 3.3 |
| TSH (mIU/L) | 2.26 |
| CK-MM (%) | 100 |
| CK-MB (%) | 0 |
| CK-BB (%) | None detected |
| CK-Total (units/L) | 107 |
| Cyclic C-Peptide IgG (units) | < 16 |
| Anaplasma Antibody IgG | Negative |
| Anaplasma Antibody IgM | Negative |
| Lyme Disease Antibody | Negative |
| HIV Antigen/Antibody | Non-reactive |
| COVID-19 | Negative |
| Influenza A & B | Negative |
| WBC (thousand/µL) | 4.7 |
| Hgb (g/dL) | 15.3 |
| Hct (%) | 46.1 |
| RBC (x10^6^/µL) | 5.48 |
| MCV (fL) | 84.1 |
| MCHC (g/dL) | 33.2 |
| MCH (pg) | 27.9 |
| RDW (%) | 12.0 |
| Platelets (thousand/µL) | 216 |
| MPV (fL) | 11.5 |
| Absolute neutrophils (cells/µL) | 2,529 |
| Absolute basophils (cells/µL) | 19 |
| Absolute eosinophils (cells/µL) | 71 |
| Urine color & appearance | Dark yellow & clear |
| Urine glucose | Negative |
| Urine bilirubin | Negative |
| Urine specific gravity | 1.030 |
| Urine pH | 5.5 |
| Urine occult blood | Negative |
| Urine nitrite | Negative |
| Urine leukocyte esterase | Negative |
| Urine bacteria | None |
| Urine hyaline casts | None |
| Urine RBC casts | None |
| Urine WBC casts | None |
| Urine squamous cells | None |
